# Trends in breastfeeding practices and mothers’ experience in the French NutriNet-Santé cohort

**DOI:** 10.1186/s13006-021-00397-x

**Published:** 2021-07-02

**Authors:** Frédéric Courtois, Sandrine Péneau, Benoît Salanave, Valentina A. Andreeva, Marie Françoise Roland-Cachera, Mathilde Touvier, Pilar Galan, Serge Hercberg, Léopold K. Fezeu

**Affiliations:** 1 Sorbonne Paris Nord University, Inserm, Inrae, Cnam, Nutritional Epidemiology Research Team (EREN), Epidemiology and Statistics Research Center - University of Paris (CRESS), SMBH-74 rue Marcel Cachin, 93017 Bobigny, France; 2 Nutritional Surveillance and Epidemiology Team (ESEN), French Public Health Agency, Sorbonne Paris Nord University, Epidemiology and Statistics Research Center - University of Paris (CRESS), 93017 Bobigny, France; 3 Public Health Department, Avicenne Hospital, AP-HP, 93017 Bobigny, France

**Keywords:** Breastfeeding duration, Exclusive breastfeeding, Total breastfeeding, Mothers’ support, Breastfeeding cessation

## Abstract

**Background:**

France has one of the lowest rates in the world regarding breastfeeding initiation and duration. Few studies have explored breastfeeding practices in France since the middle of the twentieth century, or following from initiation to cessation. The purpose of our study was to determine trends in breastfeeding over the past decades regarding public health recommendations, and to examine mothers’ perceptions about factors known to have an impact on breastfeeding support and cessation.

**Methods:**

From the NutriNet-Santé cohort, 29,953 parous women (launched in 2009 to study relation between nutrition and health), were included in the present study. Using web-questionnaires, they were asked retrospectively if they had breastfed their youngest child or not, and if so, the duration of exclusive and total breastfeeding. For those who had breastfed, we investigated their perceptions about support at initiation and during the entire breastfeeding period and reasons for breastfeeding cessation. We also asked those who did not breastfeed about their perceptions and reasons for infant formula feeding their youngest child. Analyses were weighted according to the French census data.

**Results:**

In the NutriNet-Santé cohort, 67.3% of mothers breastfed their youngest child. The proportion of breastfed children increased over the past few decades, from 55.0% (95% CI 54.3, 55.6) in the 1970s to 82.9% (82.4, 83.4) in the 2010s. Total and exclusive breastfeeding duration went from 3.3 months and 2.4 months respectively in the 1970s to 5.9 months and 3.2 months respectively in the 2010s. Most mothers felt supported at initiation and during the breastfeeding period. A reported desire to have breastfed longer than two months was 59.5%. Mothers who did not breastfeed did it by choice (64.3%). They did not feel guilty (78.2%) and did not perceive a problem not to breastfeed (58.8%), but almost half of them would have liked to have breastfed (45.9%).

**Conclusion:**

Breastfeeding duration has increased in the past decades but did not reach the public health recommendations threshold. Targets other than mothers have to be considered for breastfeeding education, like the partner and her environment, to increase breastfeeding practices.

**Trial registration:**

The study was registered at ClinicalTrials.gov (NCT03335644).

**Supplementary Information:**

The online version contains supplementary material available at 10.1186/s13006-021-00397-x.

## Background

Breastfeeding is recommended as the normal infant diet and the risks of not breastfeeding are more and more documented [1]. However, despite the World Health Organization’s (WHO) recommendation to exclusively breastfeed during the first six months of life for optimal growth, development and health, followed by continued breastfeeding along with the introduction of appropriate complementary foods for up to two years or beyond [2], too few infants are breastfed in the world. Only 40% of newborns are exclusively breastfed until the first six months of life, with higher prevalence in low-income countries compared to upper-middle-income countries [1]. Over the past decades multiple efforts have been made in many countries through public health programs, to increase breastfeeding initiation and duration. In France, breastfeeding initiation increased stepwise between 1972 (36.0%) and 1998 (52.5%) [3, 4]. One of the objectives of the French National Nutrition and Health Program, launched in 2001, was to increase breastfeeding duration and exclusive breastfeeding initiation from 55% (in 2005) to 70% in 2010 [5], in particular with a food guide intended for pregnant women [6, 7]. Although this objective was almost reached [4, 8], the prevalence of ever breastfeeding in France still remains among the lowest in the world (as Spain and the USA) in 2010 [1]. At one month, over half of infants (54%) are breastfed, and among those only 35% exclusively [9]; at six months the rates drastically fall between one in five infants being breastfed [10, 11] and one infant in four, half of them receiving complementary infant formula [12]. The median duration of total breastfeeding also has increased in France, from eight [13] to 10 weeks [14, 15] in the 1990s to 15 to 17 weeks in the early 2010s [8, 11, 12]. Despite this progress, France has not yet reached the breastfeeding duration observed in other countries, such as Sweden, Finland or Austria [16].

Breastfeeding initiation and duration depend on the context of birth and parents’ characteristics [9, 10, 17]. As breastfeeding promotion still remains a priority [18], it is important to focus on factors associated with breastfeeding initiation and duration. Thus, the purpose of the present study was to determine the trends in breastfeeding practices over decades and examine mothers’ perceptions about factors that are potential facilitators of breastfeeding support in French parous women participating in the NutriNet-Santé cohort.

## Methods

The NutriNet-Santé study is a large web-based prospective observational cohort of adult volunteers aged ≥18 years, launched in France in May 2009, with the main objective being the study of the relation between nutrition and health [19]. Briefly, the NutriNet-Santé study was implemented in the general population, targeting Internet-using adult volunteers recruited by multimedia campaigns. Using a dedicated website [20], participants were asked to complete self-administered questionnaires at baseline and every year thereafter, as well as optional questionnaires during follow-up on a monthly basis. The baseline and annual questionnaires provide information on sociodemographic characteristics and health status. Among optional questionnaires one was sent in 2014 to all women participants in the NutriNet-Santé Study; it was intended to collect information on lactation history, using the child’s health book when possible, as well as support and encouragement during breastfeeding. All participants signed an informed consent form.

Women were asked to report if they had biological children. If so, women had to give the year and month of birth for each live birth and if they had breastfed the child. In that case, they were also asked about the duration of exclusive breastfeeding (period when infant receives breast milk only, without any additional food or drink) and the duration of total breastfeeding (period of exclusive breastfeeding followed by complementary foods with continued breastfeeding up to weaning). The corresponding breastfeeding duration could be filled in days, weeks or months. Participants filled out the questionnaire in reverse chronological order, starting with the youngest child and finishing with the oldest one (maximum 5 children). Due to an increasing proportion of missing data, as gradually going back to all the siblings, we chose to use the data only for the youngest child.

Breastfeeding women were asked about their perception of support at initiation and during the overall breastfeeding period, and the reasons of breastfeeding cessation. Mothers who did not breastfeed their youngest child were also asked about the reasons for choosing bottle feeding and their perceptions of not breastfeeding.

In total, 43,135 women completed the breastfeeding questionnaire, based on closed-ended questions. Among them, 12,041 (27.6%) were nulliparous and were thus excluded from these analyses. After excluding women with incomplete information (*N* = 401), 29,953 women were eligible for analysis. Mothers who were continuing to breastfeed their child at the time of the study (*N* = 740) were not included in mother’s perceptions about breastfeeding support and reasons for breastfeeding cessation.

To improve the representativeness of our study population, probability sampling weights were computed using the 2009 Census data for the French general population regarding age distribution, educational level, occupation, presence of a child in the household, and area of residence. A sampling probability weight was attributed to each participant using the Stata complex sample design, prior to any statistical analysis. The results are presented as adjusted means and 95% confidence intervals (95% CI) computed using the standard error of the mean for continuous variables and percentages for categorical variables. Quantitative and qualitative variables were compared between mothers who breastfed and those who did not, using either Student t test or Chi square test. Linear regression models were computed using the svy: reg module of Stata. Statistical analyses were performed using Stata® 14.2 (College Station, Texas, USA). All tests were two-sided, and the significance level at 0.05 was set.

## Results

**Fig. 1:**
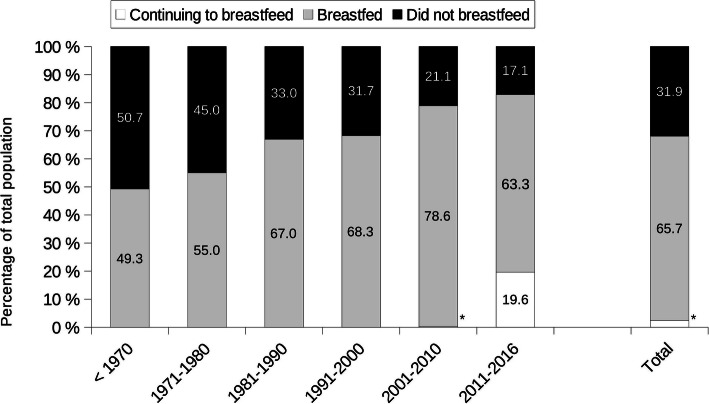
Proportion of mothers who breastfed versus did not breastfeed their youngest child through decade. Results are percentages of mothers whom last child was born in the corresponding decade. (*continuing to breastfeed: 2001–2010: 0.3%; Total: 2.4%). Children born before 1970: the median is 1967

The mean age of the 29,953 participants was 53.0 years (95% CI 52.8, 53.1) at the time of the questionnaire completion, with 39.2% aged under 50 years (Table 1). Of mothers, 67.3% breastfed their youngest child (Fig. 1). Compared to women who did not breastfeed, women who breastfed were younger at the time of the study, but older during their most recent pregnancy, they also had a higher educational level (all *p* = 0.001). Body mass index, marital status, smoking status, number of children and area of residence varied slightly but significantly among the two groups (Table 1).

**Table 1:** Characteristics of the mothers according to breastfeeding status concerning their youngest child: The NutriNet-Santé Study

Characteristics of the women	Total population	Breastfed	Did not breastfeed	***P***
***N***	29,953	20,153	9800	
**Age in years**	53.0 (52.8–53.1)	51.3 (51.2–51.5)	56.4 (56.2–56.6)	0.001
**Age during the latest pregnancy**	30.0 (29.9–30.0)	30.4 (30.3–30.5)	29.1 (29.0–29.2)	0.001
**Body mass index, kg/m**^**2**^	23.8 (23.7–23.8)	23.6 (23.5–23.6)	24.2 (24.1–24.3)	0.001
**Age categories, %**				0.001
< 40 years	19.3	23.3	11.0	
40–49.9 years	19.9	21.1	17.4	
50–59.9 years	27.1	27.0	27.1	
60 + years	33.8	28.6	44.5	
**Educational level, %**				0.001
< high school degree	21.4	15.8	32.9	
< 2y after high school degree	15.9	14.0	19.9	
≥ 2y after high school degree	62.7	70.2	47.2	
**Marital status, %**				0.001
Single	3.0	3.2	1.6	
Married	81.4	81.4	80.8	
Divorced	11.9	12.0	11.7	
Widowed	3.7	3.4	5.9	
**Smoking status, %**				0.001
Never smokers	51.3	51.6	50.7	
Former smokers	36.6	37.4	36.8	
Current smokers	13.1	11.0	12.5	
**Childbirth order, %**				0.001
1st child	24.4	24.1	24.6	
2nd child	47.0	46.0	49.8	
3rd child or more	28.6	29.8	25.6	
**Decade of childbirth, %**				0.001
< 1970	6.1	4.4	9.6	
1970–1979	19.6	15.9	27.3	
1980–1989	24.4	24.1	24.9	
1990–1999	19.7	19.8	19.5	
2000–2009	18.7	21.8	12.4	
2010–2016	11.5	14.0	6.3	
**Area of residence**^a^**, %**				0.001
Parisian region	18.7	19.1	18.0	
Paris Basin	14.8	13.4	15.6	
North	3.9	3.8	4.1	
East	8.0	8.6	6.7	
West	15.5	14.7	17.2	
South-West	11.6	11.2	12.3	
Center-East	14.8	15.6	13.2	
Mediterranean	12.7	12.6	12.9	
Overseas Regions and Departments	0.01	0.02	0.00	

^a^Research and National Development Zones (ZEAT in French): Parisian region (Île-de-France), Paris Basin (Basse-Normandie, Bourgogne, Centre-Val de Loire, Champagne-Ardenne, Basse, Haute-Normandie et Picardie), North (Hauts-de-France), East (Alsace, Franche-Comté, Lorraine), West (Bretagne, Pays de la Loire, Poitou-Charentes), South-West (Aquitaine, Limousin, Midi-Pyrénées), Center-East (Auvergne, Rhône-Alpes), Mediterranean (Languedoc-Roussillon, Provence-Alpes-Côte d’Azur, Corse), Overseas Regions and Departments (Guadeloupe, Guyane, Martinique, Mayotte, La Réunion). https://www.insee.fr/fr/metadonnees/definition/c1910

Childbirth order is the birth order of the mothers’ last child

Data are mean (95% confidence intervals computed using the standard error of the mean) for continuous variables and percentages for categorical variables

*P* values are linear trends for quantitative variables and chi square for qualitative variables

**Fig. 2:**
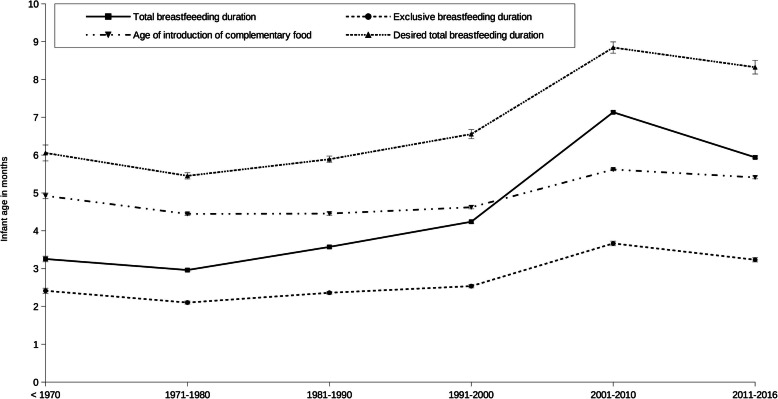
Changes in breastfeeding durations and age of introduction of complementary food. Evolution, through decades of birth of the last child, of total and exclusive breastfeeding duration, age of introduction of complementary food, and desired total breastfeeding duration. Results are mean (linearized standard error). P for trend for total and exclusive breastfeeding duration and age of introduction of complementary food = 0.001 and for desired total breastfeeding duration = 0.007

The proportion of breastfed children gradually increased with decades from 55% (95% CI 54.3, 55.6) in the 1970s to 78.9% (77.9, 80.3) in the 2000s, and 82.9% (82.4, 83.4) in the 2010s, with 19.6% continuing to breastfeed (Fig. 1). Ninety-three-point 4 % of mothers who breastfed knew the duration of total breastfeeding for their youngest child; this percentage increased with decades, from 86.1% for children born before the 1970s to 99.7% for children born in the 2010s (data not shown). Mean total breastfeeding duration was 4.7 months (95% CI 4.5, 5.0) for all decades and increased with decades, from 3.3 months (95% CI 3.1, 3.4) before the 1970s to 5.9 months (95% CI 5.6, 6.3) in the 2010s (Fig. 2A, *p*_trend_ = 0.001). On average, exclusive breastfeeding duration and the age of introduction of complementary food were 2.8 months (95% CI 2.7, 2.9) and 4.9 months (95% CI 4.9, 5.0), respectively. These rates linearly increased over the decades of study (all *p*_trend_ = 0.001, Fig. 2A). The introduction of breast-milk substitutes was concomitant with the end of exclusive breastfeeding (data not shown). The mean age of food diversification paralleled the duration of exclusive breastfeeding (Fig. 2A).

**Fig. 3:**
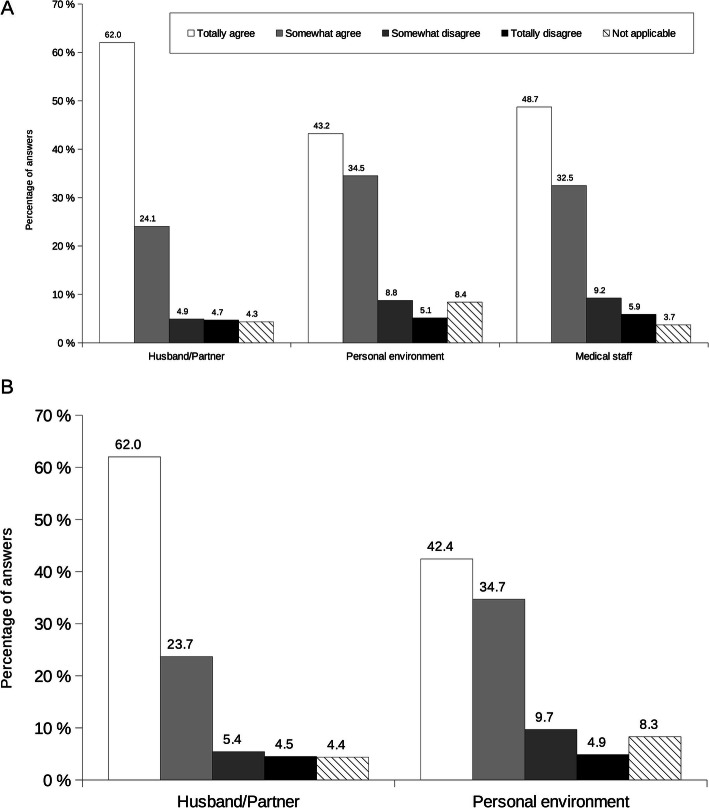
Mothers’ perceptions on (**A**) support to breastfeeding initiation and (**B**) support during breastfeeding period. Self-reported mothers’ perceptions about factors known to have an impact on support to breastfeeding. Results are percentages of answers. Mothers had choice to answer from totally agree to totally disagree for each item (see legend); a not applicable answer was possible if the item was not adapted to the mother’s life

At breastfeeding initiation, most mothers felt supported by their husband/partner (86.1% totally agreed or somewhat agreed), their personal environment (77.7% totally agreed or somewhat agreed) or the medical staff (81.2% totally agreed or somewhat agreed) (Fig. 3A). Other factors, including midwife, physician, pediatrician, Maternal and Child Protection services, lactation consultant and breastfeeding support associations, were reported as not being applicable to most mothers’ situation.

During the breastfeeding period, husband/partner and personal environment (85.7 and 77.1% totally agreed or somewhat agreed, respectively) were also sources of support for the mothers (Fig. 3B). Physician, pediatrician, lactation consultant, breastfeeding support associations and professional environment, were cited to be not applicable to most mothers’ situation.

**Fig. 4:**
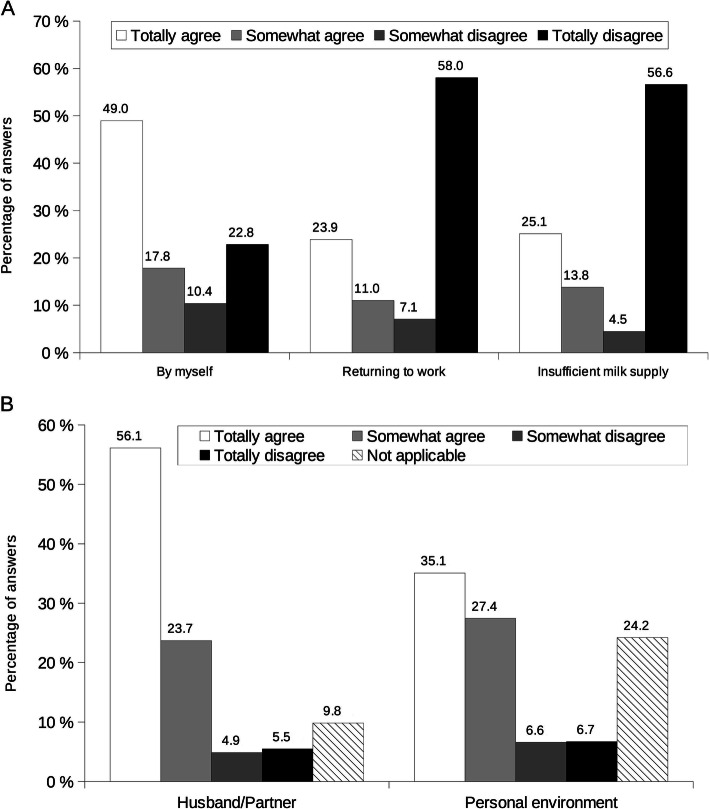
Mothers’ perceptions on (**A**) breastfeeding cessation and (**B**) support to breastfeeding cessation. Self-reported mothers’ perceptions about factors known to have an impact on (**A**) breastfeeding cessation (**B**) support to breastfeeding cessation. Results are percentages of answers. Mothers had choice to answer from totally agree to totally disagree for each item (see legend); a not applicable answer was possible if the item was not adapted to the mother’s life

Breastfeeding cessation was mothers’ choice (66.8% totally agreed or somewhat agreed, Fig. 4A); returning to work and insufficient milk supply or production were not factors felt by the mothers to have an impact on breastfeeding cessation (65.1 and 61.1% totally disagreed or somewhat disagreed respectively). Husband/partner, their personal environment, a common agreement with the husband/partner, the time commitment involved, need to take medication, nipple cracks/fissure and pain, sucking problems, being separated from the baby, breastmilk refusal by nursery and maternal assistant, breastmilk told to be “not good”, baby not wanting to be breastfed anymore, restarting smoking and fatigue/exhaustion were cited by less than 18.6% of the mothers (Additional File 1A).

When stratifying by total breastfeeding duration (< 3 months and ≥ 3 months), sucking problems, nipple cracks/fissure and pain, and insufficient milk production were substantially less likely to be reported as personal reasons for their own breastfeeding cessation (a decrease of 87.2, 81.5 and 45.0% in the percentage of mothers who totally agreed or somewhat agreed, respectively) among mothers who breastfed for more than three months compared to those who breastfed less than three months (Table 2). On the other hand, baby stopping suckling and mother returning to work were more likely to be reported as personal reasons for their own breastfeeding cessation after three months (an increase of 187.0 and 36.2% in the percentage of mothers who totally agreed or somewhat agreed respectively).

**Table 2:** Comparison of factors known to have an impact on breastfeeding cessation depending on breastfeeding duration

**Agreement to the question**	**Insufficient milk supply**		**Nipple cracks-pain**		**Sucking problems**	
	**< 3 months**	**≥ 3 months**	**< 3 months**	**≥ 3 months**	**< 3 months**	**≥ 3 months**
Totally agree	34.3	12.5	11.9	1.2	8.2	0.7
Somewhat agree	13.5	13.7	7.0	2.3	5.1	1.0
Somewhat disagree	4.2	4.9	6.1	4.2	5.8	3.5
Totally disagree	48.0	68.9	75.0	92.3	80.9	94.8
*P* values	0.001		0.001		0.001	
**Agreement to the question**	**Returning to work**		**Baby stopped suckling**			
	**< 3 months**	**≥ 3 months**	**< 3 months**		**≥ 3 months**	
Totally agree	22.3	26.5	3.7		12.1	
Somewhat agree	8.1	14.9	3.2		7.7	
Somewhat disagree	6.5	7.7	5.5		4.6	
Totally disagree	63.1	50.9	87.6		75.6	
*P* values	0.001		0.001			

Mothers were asked if some factors could have been personal reasons for their own breastfeeding cessation. Breastfeeding duration: < 3 months and ≥ 3 months

Results are percentages of answers. Mothers had choice to answer from totally agree to totally disagree for each item (see legend)

At breastfeeding cessation, mothers felt supported by their husband/partner (79.8% totally agreed or somewhat agreed) or their personal environment (62.5% totally agreed or somewhat agreed) (Fig. 4B). Physician, pediatrician, lactation consultant and breastfeeding support associations were cited to be not applicable to most mothers’ situation (Additional File 1B).

**Fig. 5:**
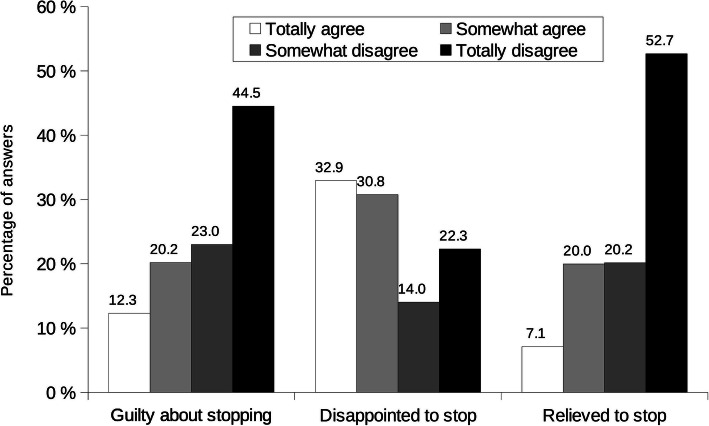
Self-reported mothers’ perceptions about stopping breastfeeding. Results are percentages of answers. Mothers had choice to answer from totally agree to totally disagree for each item (see legend); there was no possible not applicable answer as the questions were applicable to each mother

Most mothers felt neither guilty nor relieved to stop, but most of them felt disappointed that breastfeeding came to an end (Fig. 5); 59.5% reported a desire to have breastfed their youngest child longer (data not shown). Total breastfeeding duration desired by mothers was 6.9 months (95% CI 6.7, 7.27) for all decades and increased with decades, from 6.1 months (95% CI 5.6, 6.5) before the 1970s to 8.3 months (95% CI 8.0, 7.2) in the 2010s (Fig. 2B, *p*_trend_ = 0.001). This represents more than two months longer than reported total breastfeeding duration.

**Fig. 6:**
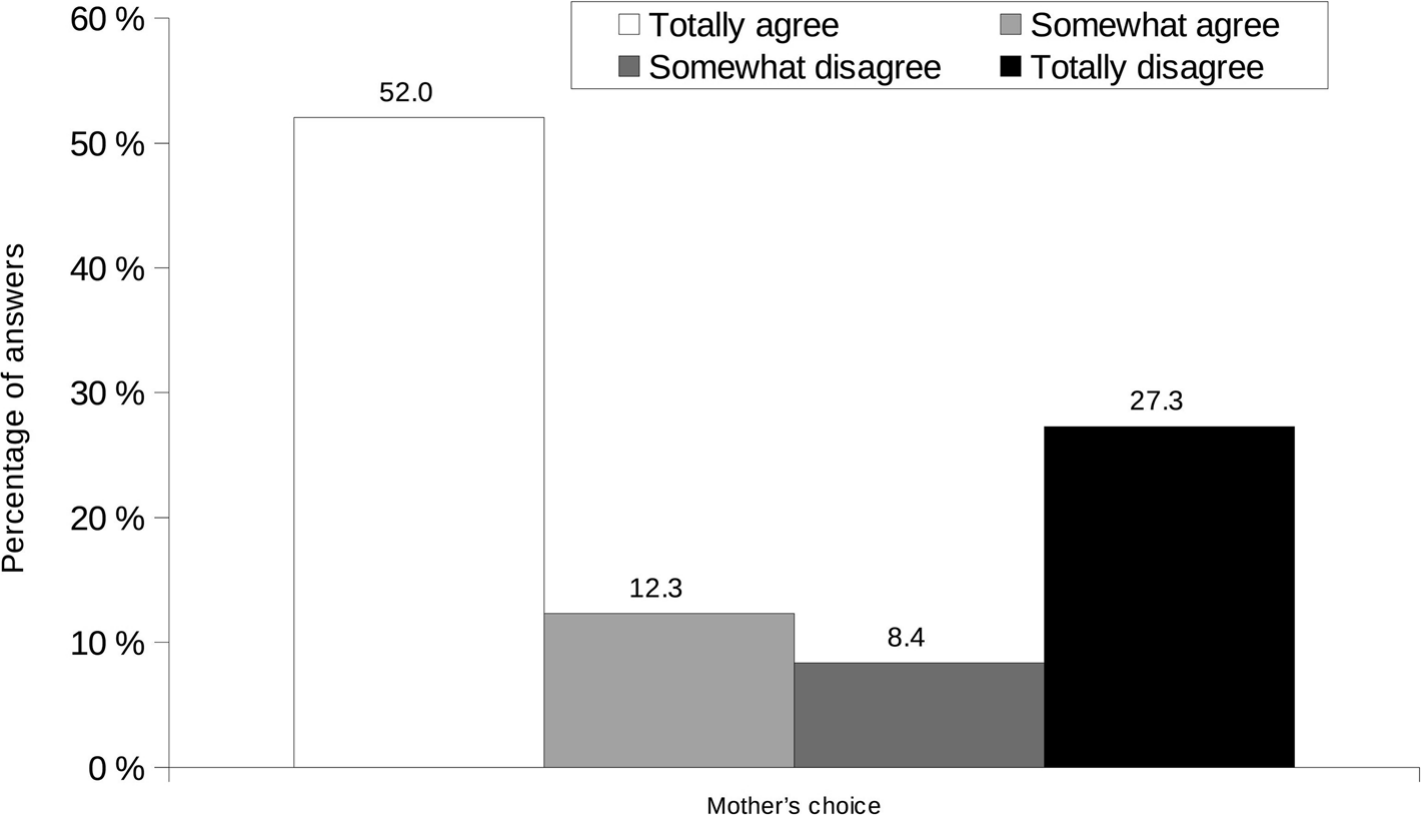
Mothers’ reason for not breastfeeding. Results are percentages of answers. Mothers had choice to answer between totally agree to totally disagree for each item (see legend); there was no not applicable answer possible as the questions were applicable to each mother

**Fig. 7:**
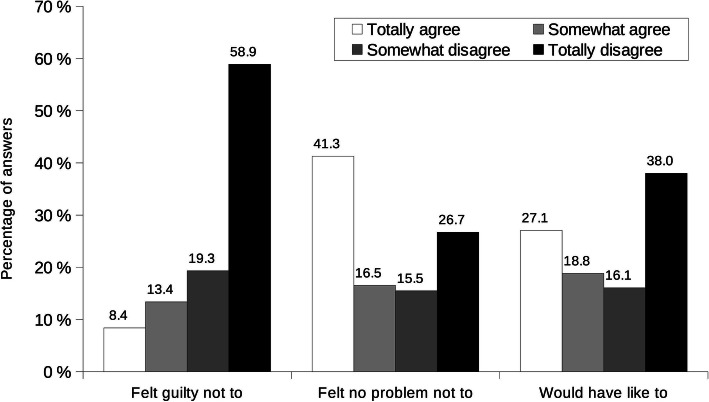
Self-reported mothers’ perceptions about not breastfeeding. Results are percentages of answers. Mothers had choice to answer from totally agree to totally disagree for each item (see legend); there was no possible not applicable answer as the questions were applicable to each mother

Among those who did not breastfeed, the majority decided not to (Fig. 6). Incitement by husband/partner, by personal environment or by a health professional, returning to work, need to take medication, mother’s or baby’s illness, childbirth complications, being separated from the baby, breastmilk told to be “not good”, insufficient breastmilk production, restarting smoking and baby not able to suckle were reported (from 84.8 to 97.5%) by these mothers not to be responsible for the decision to bottle feed their youngest child (data not shown). They did not feel guilty nor did they feel that their decision had unfavorable consequences (Fig. 7). Nevertheless, almost half of them would have liked to breastfeed their youngest child (45.9% totally agreed or somewhat agreed).

## Discussion

More than two thirds of mothers in our cohort breastfed their youngest child, thus confirming the findings in other studies in France [4, 8, 10, 17, 21]. Similar to prior research, we noted an increasing proportion of breastfed children through the decades; less than one child to two was breastfed in the 1970s (36.0% in 1972 and 45.5% in 1977) [3], four children to five was breastfed since the early 2000s (74.0% in 2012–2013) [8]. Social and demographic factors associated with breastfeeding were those frequently found in the literature: women who breastfed had a higher educational level [22], were more often non-smokers [9], and were older during the most recent pregnancy [10, 11].

Exclusive and total breastfeeding duration has increased in the last 50 years in a ratio of one to two in France. The median duration of total breastfeeding for all decades was similar to that found in other cohorts (13 weeks vs 15 to 17 weeks) [8, 11, 12]; when comparing the rates in each decade, we observed slightly higher durations, going from 13 weeks (3 months) until the 1990s (8 to 10 weeks) [13–15] to 21 weeks since the 2010s (15 to 17 weeks) [8, 11, 12]. Similarly, the median duration of exclusive breastfeeding was higher (11 weeks vs 3.4 to 7 weeks) [8, 11, 12] in our study. In the 2010s, even if we did not observe the decrease in breastfeeding percentage that the National Perinatal Survey noted in 2016 [21], a decrease in total and exclusive breastfeeding duration was seen, confirming an evolution in mothers’ behavior in this decade.

In agreement with our results at initiation and during the breastfeeding period, fathers play a significant role in the maternal decision to breastfeed [23, 24], in breastfeeding initiation [25–27], and support, decreasing the perception of milk insufficiency and therefore reducing breastfeeding interruption, especially when fathers are trained to prevent and manage the most common lactation problems [28, 29]. This high percentage of mothers being supported by their husband/partner could explain why our cohort had a higher exclusive and total breastfeeding duration compared with other cohorts in France.

Returning to work and insufficient milk production are both known to be the most common reasons for breastfeeding discontinuation and cessation, especially before four months [8, 30, 31]. In our cohort, mothers who breastfed less than three months were more likely to stop breastfeeding because of sucking problems, nipple cracks/fissure and pain, and insufficient milk production compared to those who breastfed more than three months. Moreover, insufficient milk production was more likely to be perceived as the cause of breastfeeding cessation: almost half (47.8%) of mothers who breastfed less than three months totally agreed or somewhat agreed with this versus only one fourth (26.3%) among those who breastfed more than three months. On the other hand, baby stopping suckling and mother returning to work were more cited as a cause of breastfeeding cessation by mothers who breastfed for more than three months. In France, maternity leave is 10 weeks (2.5 months) after delivery, corresponding to the exclusive breastfeeding duration (median: 2.54 months) and the moment of breastmilk substitute introduction we have observed. As it has been shown that including bottle feeding infant formula is one of the most frequent reasons for early breastfeeding discontinuation as mothers perceive that the baby prefers the bottle to the breast [32], it might be possible that exclusive breastfeeding cessation due to returning to work led to a preference for bottle feeding by the baby, thus stopping to suckle. Mothers’ own choice was most often reported as the main reason for breastfeeding cessation, and they felt supported by their husband/partner or their personal environment at that moment. This support may have played a role in the perception of not feeling guilty at that moment (67.5%). They did not seem to have stopped breastfeeding due to lassitude as they were not relieved to stop (72.8%), but approximately two thirds (63.7%) of them felt disappointed that breastfeeding had come to an end, thus leading most of them (59.5%) to the desire to have breastfed their last child more than 2 months longer. The husband/partner or the environment were not cited to have played a role in breastfeeding cessation, but it was interesting to see that even if the main reason was the mother’s own choice, they nevertheless would have liked to breastfeed longer. We could have compared this choice to the fact that mothers felt it was time to stop breastfeeding, but this matches to mothers with eight months duration and over [8]. As most mothers did not reach WHO’s recommendations for exclusive and continued breastfeeding, this choice might be influenced by other factors that have to be determined.

Most mothers, who did not breastfeed their youngest child, made the decision on their own; they did not feel guilty and, in a lesser extent, felt no problem not to breastfeed the child. Even if they were not incited to bottle-feed by their husband/partner, nor by their personal environment, it has been shown that the fathers have misperceptions and a lack of education about breastfeeding, and are more likely to think that it is bad for the breasts, makes the breasts ugly and interferes with sex [33].

Despite this, almost half of those mothers would have like to breastfeed their youngest child. These findings show for the first time that some non-lactating mothers have ambivalent perceptions regarding breastfeeding. Public health programs may play a role in people’s perception of breastfeeding and the risks of not breastfeeding.

One limitation of our study is that this cohort of mothers was not representative of the overall population in France. Compared to other cohorts, our mothers had higher educational level, were mostly multiparous and had higher total and exclusive breastfeeding durations, but they were of mostly the same age [8, 9, 11, 12]. Another limitation is the memory bias, as some mothers had a recall period up to 50 years when they did not have access to the child’s health book as recommended in our questionnaires. Thus, breastfeeding duration might have been underestimated with a recall period of eight or more years and overestimated with a recall period of 14 to 15 years [34]. Social desirability bias could also have played a role, as it has been demonstrated that mothers who breastfed on a short duration tended to overreport, while those who breastfed on long durations underreported [35]. Nevertheless, recall accuracy does not differ over recall periods of 34–50 years [35]; the decline in recall accuracy seems to happen in the first years after delivery [36]. According to Promislow et al., the misclassification in breastfeeding duration after 34–50 years is comparable to this “found for nutrients when comparing food frequency questionnaires with diet records”. That’s why maternal recall still remains the standard for large scale epidemiological surveys [37].

## Conclusions

A great improvement in breastfeeding initiation has been shown in France in the past decades, thanks to public health programs such as the French Nutrition and Health Program. However, even if we have observed that breastfeeding duration (exclusive and total) has increased over decades in this study, the objective of exclusive breastfeeding during the first six months of life, followed by continued breastfeeding with appropriate complementary foods for up to two years or beyond is far from being achieved. More worrying is the fact that this tendency seems to be unstable as exclusive and total durations of breastfeeding of children born in the 2010s, have started to decline. The results of this study emphasize the need to keep working on how to reach this objective and maybe find other targets apart from the mothers [38], as the fathers seem to play a major role and also well-trained health professionals, in order to prevent sucking problems, nipple cracks/fissure and pain, and insufficient milk production. Increasing the duration of the maternity leave and reducing working time to part-time during the first year must be set up to increase breastfeeding duration [39]. Further research is needed to evaluate fathers’ impact, and other determinants, on breastfeeding durations.

## Supplementary Information

**Additional file 1.** Mothers’ perceptions on (A) breastfeeding cessation and (B) support to breastfeeding cessation. Self-reported mothers’ perceptions about factors known to have an impact on (A) breastfeeding cessation (B) support to breastfeeding cessation, but that were not felt by mothers to have played a role. Results are percentages of answers. Mothers had choice to answer from totally agree to totally disagree for each item (see legend); a not applicable answer was possible if the item was not adapted to the mother’s life.

## Data Availability

The datasets used and/or analysed during the current study are available from the corresponding author on reasonable request.

## Abbreviation

WHO: World Health Organization
