# Supplementary figures and images for: Trends in breastfeeding practices and mothers’ experience in the French NutriNet-Santé cohort
Source: Int Breastfeed J. 2021 Jul 2;16:50. doi: 10.1186/s13006-021-00397-x (PMC8254215; doi:10.1186/s13006-021-00397-x)

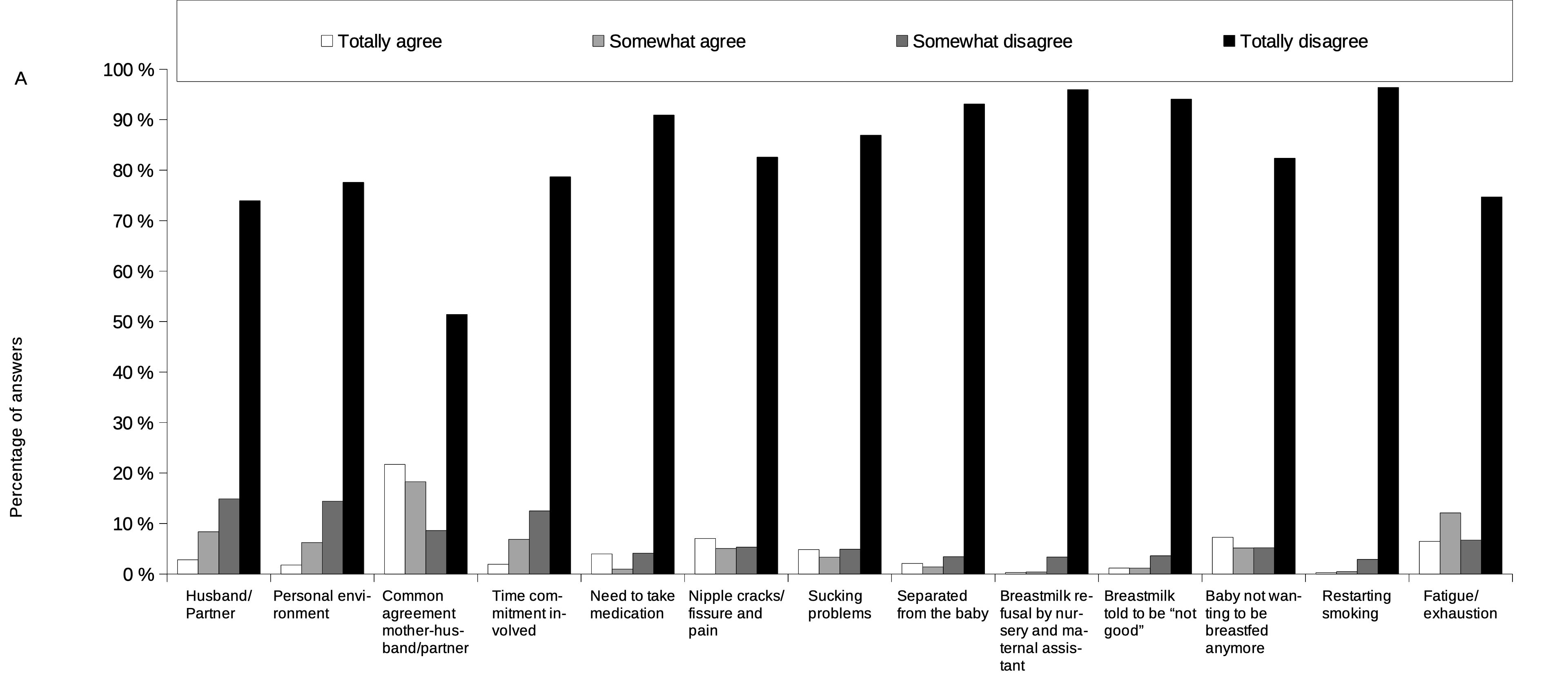

Supplement: Supplementary file 1 — Additional file 1. Mothers’ perceptions on (A) breastfeeding cessation and (B) support to breastfeeding cessation. Self-reported mothers’ perceptions about factors known to have an impact on (A) breastfeeding cessation (B) support to breastfeeding cessation, but that were not felt by mothers to have played a role. Results are percentages of answers. Mothers had choice to answer from totally agree to totally disagree for each item (see legend); a not applicable answer was possible if the item was not adapted to the mother’s life. [file 13006_2021_397_MOESM1_ESM.zip › SM Figure 1A.jpg]

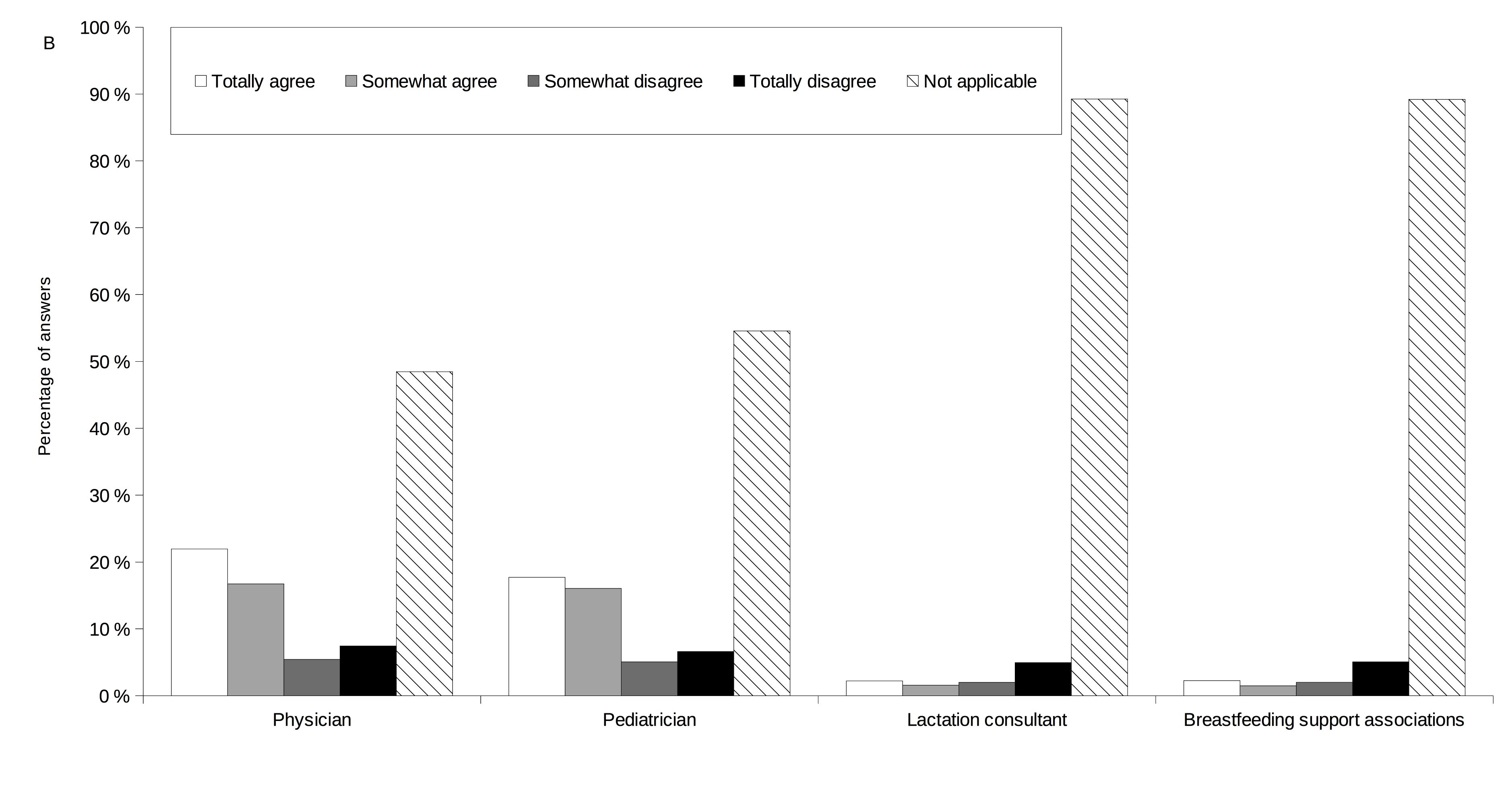

Supplement: Supplementary file 1 — Additional file 1. Mothers’ perceptions on (A) breastfeeding cessation and (B) support to breastfeeding cessation. Self-reported mothers’ perceptions about factors known to have an impact on (A) breastfeeding cessation (B) support to breastfeeding cessation, but that were not felt by mothers to have played a role. Results are percentages of answers. Mothers had choice to answer from totally agree to totally disagree for each item (see legend); a not applicable answer was possible if the item was not adapted to the mother’s life. [file 13006_2021_397_MOESM1_ESM.zip › SM Figure 1B.jpg]
